# Poor adherence to cancer therapy in Ethiopia: systematic review and meta-analysis

**DOI:** 10.3389/phrs.2026.1608819

**Published:** 2026-06-25

**Authors:** Astewle Andargie Baye, Yirgalem Abere, Demewoz Kefale, Yeshiambaw Eshetie, Mengistu Ewunetu, Lakachew Yismaw Bazezew, Gebrie Kassaw Yirga, Gebrehiwot Berie Mekonnen

**Affiliations:** 1 Department of Adult Health Nursing, College of Health Science, Debre Tabor University, Debre Tabor, Ethiopia; 2 Department of Neonatal Nursing, College of Health Sciences, Debre Tabor University, Debre Tabor, Ethiopia; 3 Department of Pediatrics and Child Health Nursing, College of Health Sciences, Debre Tabor University, Debre Tabor, Ethiopia

**Keywords:** adherence, adverse effects, cancer therapy, comorbidity, oncology

## Abstract

**Objectives:**

Poor adherence significantly compromises the effectiveness and success of cancer treatment. Understanding the full scope and contributing factors of poor adherence is essential for improving patient care. Therefore, this review aimed to determine the pooled prevalence of poor adherence to cancer therapy in Ethiopia.

**Methods:**

The preliminary concepts were registered into PROSPERO. Comprehensive searches of multiple databases were conducted to identify relevant articles. A random-effects model was used to estimate the pooled effect size. Heterogeneity was assessed using the I^2^ statistic. Publication bias was evaluated through both qualitative and quantitative methods. Additionally, a sensitivity analysis was done to ensure the robustness of the studies.

**Results:**

The analysis includes 15 studies with a total of 7,115 cancer patients. The result indicates that, the overall pooled prevalence of poor adherence to cancer therapy in Ethiopia was 41.45% (95% CI: 33.37–49.52). Comorbidity, treatment side-effects, and residency settings are factors independently associated with poor adherence.

**Conclusion:**

Poor adherence to cancer therapy in Ethiopia is significant. Therefore, efforts are needed to increase adherence. Comprehensive and timely management of comorbid conditions and treatment adverse effects can increase adherence to treatment.

## Introduction

Cancer is a major worldwide public health concern. It is the leading cause of morbidity and mortality [1]. Globally, in 2022, there were 20 and 9.7 million new cases of cancer and death from the disease respectively [2]. Ethiopia is among the developing countries where the burden is growing. According to recent data from the Global Cancer Observatory and the International Agency for Research on Cancer (IARC), 80,334 new cases of cancer and 54,698 deaths occurred in Ethiopia [3].

The primary goals of cancer treatment are to achieve cure, prolong life, alleviate suffering, and control disease metastasis [4, 5]. As a result, cancer patients are required to follow their treatment plans consistently without interruption [6]. However, practically, they are often not having regular follow-up [7]. Most cancer patients had poor adherence to their treatment plans [8]. Non-adherence to cancer treatment remains a serious and growing problem [9]. According to, World Health Organization (WHO) adherence is defines as the degree to which a patient’s behavior, taking medication, following a diet, and/or executing lifestyle changes corresponds with agreed recommendations from a healthcare provider [10]. Inadequate adherence to cancer therapy occurs when patients do not follow their prescribed treatment plans consistently or as directed by their healthcare providers, either intentionally or unintentionally [11].

Several barriers and risk factors have been associated with poor adherence [12]. Studies have shown that advanced age, being unmarried, lack of social support, and the high cost of treatment negatively impact adherence to therapy [13, 14]. Additionally, inadequate communication between patients and healthcare providers is linked to poor adherence [13, 15, 16]. Other factors include forgetfulness, and unrealistic beliefs or perceptions about cancer and its treatment are also associated with lower levels of adherence [17, 18]. Furthermore, comorbidities and adverse drug effects are described as the most obvious causes of non-adherence behavior [19, 20]. Approximately, 25% of cancer patients are intentionally interrupt their anti-cancer treatment in relation to drugs side effects [21].

The effectiveness and success of cancer treatment are significantly compromised by poor adherence. Evidence indicates that cancer patients with poor adherence experienced adverse health outcomes, including increased morbidity, decreased survival rates, and reduced quality of life [22]. Scholars emphasizes that addressing poor adherence requires a multifaceted approach; no single intervention is sufficient. Commitment from patients, healthcare providers, and patient advocates is essential [23, 24]. Ethiopia, has implemented national cancer control programs and strategies to reduce cancer burden by identifying and minimizing risk factors, as well as promoting screening, early diagnosis, and treatment [25]. However, ensuring cancer patients adherence to their treatment plans remains one of the country’s major challenges. Understanding the full scope and contributing factors of poor adherence is essential for improving patient care. Therefore, this review aims to determine the pooled prevalence of poor adherence to cancer therapy in Ethiopia.

## Methods

### Registration and reporting protocols

This review was registered in the PROSPERO database with protocol ID CRD42025637788. It was conducted based on the Preferred Reporting Items for Systematic Reviews and Meta-Analyses (PRISMA) guidelines.

### Search strategies

A comprehensive search was conducted across multiple sources, including PubMed, the African Journals Online (AJOL), and CINAHL (EBSCO). To ensure the inclusion of additional relevant studies not indexed in electronic databases, we extensively searched search engines such as Google and Google Scholar. Additionally, Ethiopian university institutional research repositories were explored to identify unpublished studies. The searches of electronic databases and engines were performed systematically, utilizing appropriate Medical Subject Headings (MeSH) terms. For each topic, search terms were combined with the Boolean operator “OR,” while different concepts were combined using “AND.” The following key terms and phrases have been used: (Poor [All Fields] AND adherence [All Fields]) OR (adherence [All Fields] AND (“neoplasms [MeSH Terms] OR “neoplasms [All Fields] OR “cancer [All Fields])) OR (“neoplasms [MeSH Terms] OR “neoplasms [All Fields] OR “oncology [All Fields]) OR (“therapy [Subheading] OR “therapy [All Fields] OR “treatment [All Fields] OR “therapeutics [MeSH Terms] OR “therapeutics [All Fields]) OR ((“therapy [Subheading] OR “therapy [All Fields] OR “therapeutics [MeSH Terms] OR “therapeutics [All Fields]) AND (“Ethiopia [MeSH Terms] OR “Ethiopia [All Fields])) (Sf1).

### Eligibility criteria

All studies conducted in Ethiopia that reported on adherence to cancer therapy regardless of age, cancer stage, phenotype, primary location, or treatment statuses are included. Only studies published in English were considered. Conversely, research that did not quantitatively address the outcome of interest, such as narrative studies, systematic reviews, meta-analyses, and animal experiments, was excluded.

### Outcome measurement

The primary outcome interest of this review was to identify poor adherence to cancer therapy.

Every study included in this review and analysis met the PICOS/PECOS criteria listed below:

Population/participant: All peoples diagnosed as having cancer.

Intervention/Exposure: Any factors that impacts adherence to cancer therapy.

Comparator Good adherent cancer patients.

Outcome: Poor adherence to cancer therapy in Ethiopia.

Study type: All observational studies.

### Study screening

Two independent authors reviewed the search results from each database (AAB & YA). The process was carried out in four stages. First, all eligible articles were downloaded and organized using reference management software such as EndNote X7. In the second stage, the articles were sorted based on their titles and abstracts, with irrelevant results and duplicates removed. The third phase involved assessing each study’s eligibility according to predefined inclusion and exclusion criteria. Finally, the authors compared their screening outcomes, and any discrepancies were resolved through discussion with a third author (GBM).

### Quality appraisal

Eligible studies were evaluated for final inclusion. The methodological quality of each study was assessed using the standardized Joanna Briggs Institute (JBI) critical appraisal checklist. For each checklist item, four response options were available: yes, no, unclear, and not applicable. A score of one (1) or zero (0) was assigned to each item, with studies that did not meet the inclusion criteria for a particular item receiving a score of zero and those that satisfied the criteria receiving a score of one (1). The scores for all items were summed and converted into a percentage. Studies were then categorized based on their overall score: poor or low quality if less than 50%, good quality if between 50% and 75%, and high quality if 75% or above. Only articles of good quality and above were included for evidence synthesis and interpretation. The overall assessment result has shown that, two studies fall under good quality, the rest were with high quality and all were rated as having a low risk of bias. The overall evidence risk of bias suggests studies employed robust methodologies, support the reliability of the overall findings and conclusion. In addition, the certainty of evidence, as determined by GRADE (Grading of Recommendations Assessment, Development and Evaluation) approach, suggests that the true effect is close to the estimated effect (Sf 2).

### Data extraction

Full data extraction from the included studies was performed independently by three authors. The extracted information included author names, publication year, study design, sample size, effect size, and participant age. A standardized data abstraction form was used to ensure consistency. Any discrepancies among the authors were resolved through discussion, with guidance from a senior researcher.

### Statistical analysis

Stata version 17 was used for the final data analysis. We have used a random effects model to estimate pooled effect size. The overall pooled estimate was presented using a forest plot. Heterogeneity among studies was assessed with the I^2^ statistic. To address heterogeneity, subgroup analyses were conducted. Sensitivity analyses were performed to evaluate the influence of individual studies on the pooled estimate. Publication bias was examined qualitatively through the funnel plot and quantitatively using Egger’s regression test. A p-value greater than 0.05 from Egger’s test indicates the absence of small study effects.

## Results

### Search results

**FIGURE 1:**
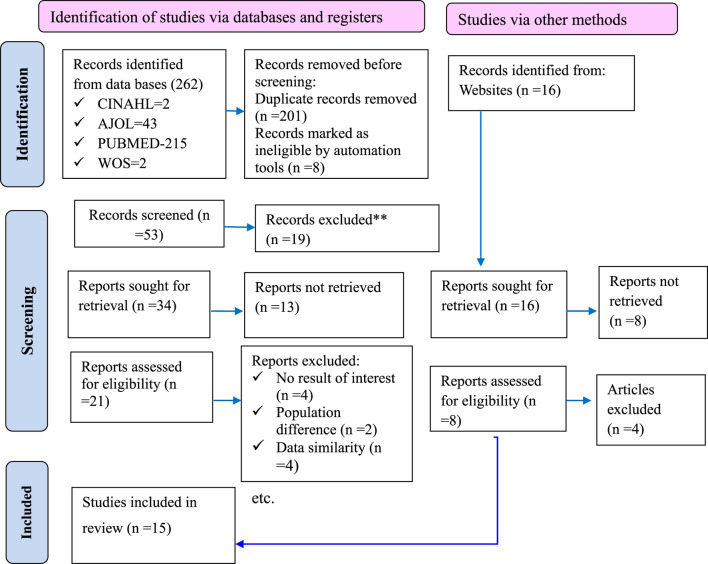
Diagram PRISMA flow showing the steps for screening the articles, Ethiopia [2025].

A total of 278 articles were identified from various databases and sources. After removing duplicates, 69 articles remained for screening. Reports from 50 articles were retrieved, but 21 were excluded based on eligibility criteria. The full texts of 29 articles were then assessed for inclusion. Following a review of their titles and abstracts, 15 articles were selected for final synthesis (Figure 1).

### Characteristics of the included studies

This review encompasses a total of 15 studies [8, 15, 26–37]. Of these, eight were conducted in Addis Ababa [15, 26, 30–32, 35–37], three in the Amhara region [8, 34, 38], two in Oromia [27, 29], and two involved both Addis Ababa and Oromia [29, 33]. Overall, these studies included 7,115 cancer patients. The participants’ ages ranged from 7.2 to 49 years [29]. Female patients comprised about 80.25% of the sample. Among the 1,501 female cancer patients about 65% were in the post-menopausal age range [15, 27, 30, 31, 37]. The majority(73.4%) of patients were married [8, 15, 26–28, 30, 31, 33–35, 37, 38]. Literacy levels were reported in nine studies, revealing that 66.4% of participants can read and write [8, 15, 26–28, 30, 33, 38]. Out of 2,960 cancer patients, 60% are from rural areas [8, 15, 26, 27, 30, 34, 35, 37]. Additionally, approximately 70% of patients traveled more than 100 km to access treatment facilities [8, 15, 26, 30, 31, 35]. Cervical cancer was the most common cancer phenotype, accounting for 46.65% of cases. Breast cancer was the second most prevalent, representing 15.7%, followed by hematological cancers at 9%. Other noted types included colorectal cancer (3.4%), head and neck cancers (2.3%), and ovarian and uterine cancers (2.2%). A significant majority (71.5%) of patients were diagnosed at an advanced stage of cancer [8, 15, 27, 30, 32, 34, 36, 37]. Regarding comorbidities, 13.3% of 4,141 cancer patients were HIV-positive [8, 15, 26, 28, 36, 37]. Furthermore, among 2,162 patients, 68% experienced treatment-related adverse effects [8, 15, 26, 30, 31, 37, 38] (Table 1).

**TABLE 1:** Table of Summary characteristics of the included studies, for adherence study, Ethiopia [2025].

Author (Year)	Study region	Study design	Mean age	Prevalence	Cancer type	Treatment Type	Quality score
Moelle et al. [37]	Addis Ababa	Cohort	49	34	Cervical	Radiotherapy	62.5%
Mulu Fentie et al. [26]	Addis Ababa	Cross-sectional	37.8	44.9	Leukemia	Chemotherapy	87.5%
Reibold et al. [27]	Oromia	Cohort	45	65	Breast	Hormonal	75%
Stroetmann et al. [28]	Both	Cross-sectional	34	55.3	Cervical	Others	75%
Hassen et al. [30]	Addis Ababa	Cross-sectional	41.99	16.4	Breast	Chemotherapy	87.5%
Gebre et al. [31]	Addis Ababa	Cross-sectional	NR	30.3	Cervical	Combination	87.5%
Alemayehu et al. [33]	Both	Cross-sectional	34	45.8	Cervical	Others	87.5%
Kibret et al. [34]	Amhara	Cross-sectional	48.5	21.4	Mixed	Combination	87.5%
Bekalu et al. [8]	Amhara	Cross-sectional	48	57.7	Mixed	Chemotherapy	87.5%
Wako et al. [15]	Addis Ababa	Cross-sectional	NR	41	Breast	Hormonal	87.5%
Rick et al. [36]	Addis Ababa	Cross-sectional	48	24	Mixed	Radiotherapy	62.5%
Feuchtner et al. [32]	Addis Ababa	Cohort	NR	65	Mixed	Combination	75%
Hordofa et al. [29]	Oromia	Cross-sectional	7.2	42	Mixed	Combination	87.5%
Lingerih et al. [35]	Addis Ababa	Cross-sectional	10	52.9	Bone tumors	Combination	87.5%
Degu and Kebede [38]	Amhara	Cross-sectional	44.53	32.7	Breast	Chemotherapy	87.5%

NR = Not recorded.

### Poor adherence to cancer therapy

**FIGURE 2:**
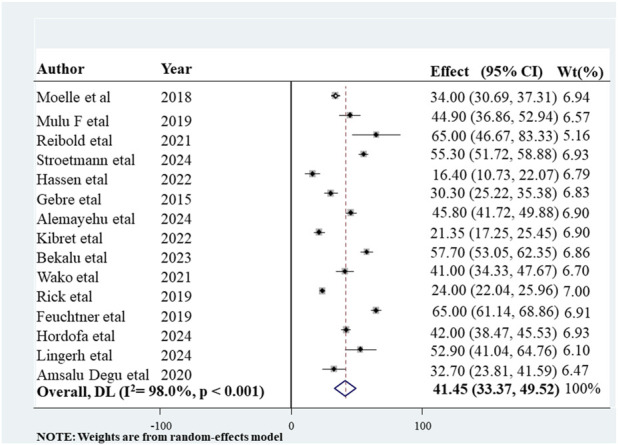
Figure of forest plot of the pooled prevalence of poor adherence among cancer patients in the Ethiopia, [2025].

In Ethiopia, the crude prevalence of poor adherence to cancer treatment ranges from 16.4% to 65% [32, 36]. Meta-analysis results indicate the overall pooled prevalence of poor adherence was 41.45% (95% CI: 33.37–49.52). The statistical test (I^2^_=_98.0%, P < 0.001) reveals a significant degree of heterogeneity among the studies, indicating the need for further investigation. To address this heterogeneity, researchers recommend conducting subgroup or meta-regression analyses based on study-level characteristics [39] (Figure 2).

### Handling of heterogeneity

#### Sub-group analysis

We conducted a subgroup analysis to explore potential sources of heterogeneity based on different covariates including geographical regions, cancer phenotypes, and treatment regimens. Regarding geographical areas, in Addis Ababa the pooled prevalence of poor adherence was 38.36% (95% CI: 26.66–50.06) [15, 26, 30–32, 35–37]. Oromia region exhibited the highest pooled estimate, approximately 51.67% (95% CI: 29.42–73.92) [27, 29]. Regarding cancer phenotypes, the highest prevalence of poor adherence was observed on cervical cancer patients approximately 41.40% (95% CI: 30.13–52.18) [28, 31, 33, 37]. In addition, the pooled prevalence among breast cancer patients was 37.19% (95% CI: 20.80–53.57) [15, 27, 30, 38].

Regarding cancer treatments, the pooled prevalence of poor adherence to radiotherapy was 28.91% (95% CI: 19.11–38.71) [36, 37]. Poor adherence to chemotherapy was 37.96% (95% CI: 27.03–58.89) [8, 26, 30, 38]. Additionally, the overall prevalence of poor adherence to combination therapy was 42.08% (95% CI: 28.26–55.91), which is the highest among groups [29, 31, 32, 34, 35] (Table 2).

**TABLE 2:** Table of subgroup analysis summary on the prevalence of poor adherence to cancer therapy in Ethiopia [2025].

Covariates	Groups	No of studies	Total cancer Patients	Frequency	Crud prevalence	Group estimate	95% CI	Heterogeneity (I^2^, τ^2^, p value)
Study region	Addis Ababa	8	4101	1398	34.08	38.36	26.66–50.06	98.30, 274.6, P < 0.001
	Oromia	2	775	398	51.40	51.67	29.42–73.92	82.80, 493.4, P = 0.016
	Both	2	1315	673	51.2	50.60	41.29–59.91	91.50, 41.3, P = 0.001
	Amhara	3	924	367	39.72	37.28	11.89–62.67	98.50, 219.1, P < 0.001
Cancer phenotype	Breast	4	506	164	32.4	37.19	20.80–53.57	93.60, 250.8, P < 0.001
	Cervical	4	2417	1036	42.9	41.40	30.13–52.18	97.00, 128.0, P < 0.001
	Bone tumors	1	68	36	52.9	-	41.03–64.76	-
	Mixed	5	3,977	1467	36.9	41.97	24.99–58.96	99.02, 371.9, P < 0.001
	Blood	1	147	66	44.9	-	36.85–52.94	-
Treatment type	Chemotherapy	4	851	378	44.4	37.96	27.03–58.89	97.70, 443.4, P < 0.001
	Radiotherapy	2	2,611	706	27.04	28.91	19.11–38.71	92.29, 48.07, P < 0.001
	Combination	5	2103	910	43.27	42.15	25.53–58.78	98.40, 349.1, P < 0.001
	Hormonal	2	235	102	43.4	51.42	28.10–74.73	82.8, 238.4, P = 0.016
	Others	2	1315	673	51.2	50.60	41.29–59.91	91.50, 41.2, P = 0.001

#### Sensitivity analysis

We have conducted inverse variance weighted method sensitivity analysis to evaluate the robustness of the findings. The result of the forest plot reveals that no study-specific estimates are markedly dispersed, indicating robustness in the overall findings (Sf_3_).

#### Assessment of publication bias

**FIGURE 3:**
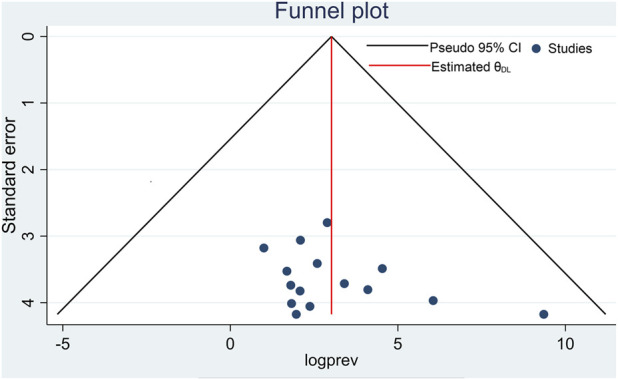
Figure of Meta funnel plot of the poor adherence to cancer therapy in the Ethiopia [2025].

The qualitative analysis of the funnel plot suggests the presence of publication bias (Figure 3). However, the P-value from the regression-based Egger test for funnel plot asymmetry is 0.1949, which exceeds the 0.05 cutoff points. This indicates no evidence of publication bias and small study effect.

#### Factors associated with poor adherence to cancer therapy

According to this study, various factors are associated with poor adherence. The likely hood of being non-adherent was observed among cancer patients with advanced stages and metastasis [34]. Additionally, the presence of comorbid conditions significantly impacts patients’ ability to follow their treatment plans. Specifically, cancer patients with comorbidities are approximately twice as likely to be poorly adherent to therapy compared to those without comorbidities (OR = 2.01, 95% CI: 1.02–3.00) [8, 15, 38]. Regarding patient-related factors, cancer patients with lower socioeconomic status and financial difficulties are particularly vulnerable to poor adherence to treatment regimens [26, 27]. Other significant factors including forgetfulness [31], limited knowledge about the disease and treatment, being unmarried [34], and unemployment are increase the risk of poor adherence [26]. Additionally, female gender, a family history of cancer, and inadequate social support are strongly associated with poor adherence [8]. Rural cancer patients also tend to be less adherent to their treatment protocols compared to their urban counterparts [15, 26]. The pooled analysis indicates, cancer patients in rural areas are 0.58 times less likely to adhere to their treatments compared to urban residents (OR = 0.58, 95% CI: 0.17–0.98). In relation to treatment-related factors such as adverse drug effects is the primary cause of non-adherence behavior [8, 15, 26, 31]. The pooled data indicate that cancer patients experiencing adverse effects are about twice as likely to be poorly adherent (OR = 2.23, 95% CI: 1.01, 3.46). From a healthcare system perspective, underdeveloped infrastructure and inadequate communication between patients and providers present significant barriers to adherence in Ethiopia [15, 27] (Table 3).

**TABLE 3:** Table of pooled analysis of factors associated with poor adherence, Ethiopia [2025].

Covariates	Studies	AOR	95% CI	Weight (%)	Pooled estimate(95% CI)	Heterogeneity (I^2^, p-value)
Comorbidity	[15]	1.60	1.19, 2.01]	43.02	2.01 [1.02, 3.00]	86.46%, p < 0.001
	[8]	0.36	−1.83, 2.55,	14.20		
	[38]	2.97	2.55, 3.39]	42.78		
Treatment adverse effect	[26]	0.16	[−1.81, 2.13]	17.35	2.23 [1.01, 3.46]	84.20%, p < 0.001
	[8]	3.37	[2.69, 4.31]	51.34		
	[31]	3.02	2.17, 3.88]	27.23		
	[15]	1.50	0.72, 2.28]	27.86		
Rural residency	[15]	0.67	[0.21, 1.13]	76.18	0.58 [0.17, 0.98]	0.00%, p = 0.01
	[26]	0.29	[−0.54, 1.12]	23.82		
		1.59	[1.22, 1.96]	28.71		

## Discussion

This review highlights about non-adherence to cancer therapy as a substantial problem in Ethiopia. The statistical analysis indicates that the pooled prevalence of poor adherence to cancer therapy in Ethiopia was 41.45% (95% CI: 33.37–49.52). This finding is nearly consistent with a study conducted in Uganda where the prevalence of poor adherence to cancer therapy was 45% [40]. However; it is significantly higher than those of the studies conducted in South Africa, Canada, Brazil, and India, where the magnitude of poor adherence in these settings was 32.7%, 27%, 9.9%, and 12.8%, respectively [24, 41–43]. The potential variation may arise from the differing healthcare infrastructures and resources available in these countries. In Ethiopia, there may be limited access to essential medications, insufficient patient education, poor communication between patients and healthcare professionals, and a lack of support systems to facilitate adherence to treatment plans [8, 15, 26]. Additionally, unmanaged treatment side effects, high cost of drugs, personal beliefs about cancer, psychological issues such as anxiety, and depression may contribute to increased poor adherence rates in Ethiopia [31, 34]. Experience in low- and middle-income countries suggests that, implementing holistic patient education, early management of treatment adverse effects and digital health strategy could increase adherence level [44, 45]. This review demonstrated that there is a significant association between comorbidity and poor adherence to cancer therapy. Cancer patients with comorbid diseases were two times more likely to be poor adherent to cancer therapy. This finding is consistent with studies from the USA [46]. Research finding revealed cancer patients often face complex health challenges, especially when they have comorbidities [47]. Evidence also shows that the presence of comorbidity increases the risk of drug-drug and disease-drug interactions and toxicities, which could adversely impact adherence to cancer treatment [48]. Prevention and early intervention of comorbidities can play a crucial role in facilitating better adherence to cancer therapy.

Another finding of this review reveals, cancer patients living in rural areas were 0.58 times less likely to adhere to their cancer treatment. This result consistent with a study conducted in Nigeria, where geographical barriers pose an obstacle to adherence in cancer therapy [49]. This might be due to cancer patients who live in rural areas are obliged to travel long distances to reach a cancer treatment center. The presence of poor infrastructure, high treatment costs, and poor perception to cancer can also lead to poor adherence.

The findings of this study also showed that treatment side effects are a significant predictor of poor adherence to cancer therapy. Adherence to cancer therapy is negatively impacted by adverse effects of treatment regimens [50, 51]. Evidence from the United kingdom indicates that patients who experience treatment side effects are more likely to discontinue their treatment [52]. This indicates that addressing treatment side effects is paramount for improving adherence rates.

### Conclusion and recommendations

Poor adherence to cancer therapy in Ethiopia is significant as compared with other findings. Therefore scholars, legislators, program managers, patient advocates, the Ministry of Health, and healthcare workers need to collaborate to increase cancer patients’ treatment adherence. The integration of routine information and education related to cancer is essential for improving adherence. Guidelines, policies, and frameworks focusing to increase adherence needs to be established. Comprehensive and timely management of comorbid conditions and treatment adverse effects can increase adherence to treatment.
