# Supplementary material for: Poor adherence to cancer therapy in Ethiopia: systematic review and meta-analysis
Source: Public Health Rev. 2026 Jun 25;47:1608819. doi: 10.3389/phrs.2026.1608819 (PMC13345979; doi:10.3389/phrs.2026.1608819)
Supplement: Supplementary file 1 [file Table1.docx]

**Database and other sources of search strategy on poor-adherence to cancer therapy in Ethiopia**

| **Data base** | **Search strategy** | **Number of articles** |
| --- | --- | --- |
| **PubMed** | (Non [All Fields] AND adherence [All Fields]) OR (adherence[All Fields] AND ("neoplasms"[MeSH Terms] OR "neoplasms"[All Fields] OR "cancer"[All Fields])) OR ("neoplasms"[MeSH Terms] OR "neoplasms"[All Fields] OR "oncology"[All Fields]) OR ("therapy"[Subheading] OR "therapy"[All Fields] OR "treatment"[All Fields] OR "therapeutics"[MeSH Terms] OR "therapeutics"[All Fields]) OR (("therapy"[Subheading] OR "therapy"[All Fields] OR "therapeutics"[MeSH Terms] OR "therapeutics"[All Fields]) AND ("ethiopia"[MeSH Terms] OR "ethiopia"[All Fields])- | 215 |
| **Web of science** | Non-adherence to cancer therapy or treatment in Ethiopia | 2 |
| **African Journals Online (AJOL)** | Adherence or Non-adherence to cancer or oncology or neoplasm therapy or treatment in Ethiopia | 43 |
| **CINAHL/EBSCO** | (MH "Adherence" OR MH "Compliance") AND (MH "Neoplasms" OR "cancer therapy") AND (Ethiopia) | 2 |
| **Grey Literature;**  **Google &**  **Google Scholar** | Magnitude of or prevalence of poor adherence to cancer therapy or treatment in Ethiopia | 16 |

**NB: Study filtration was based on observational Study, Humans, English language, and Ethiopia. The last search date was April 2/2025**
