# Supplementary figures and images for: Poor adherence to cancer therapy in Ethiopia: systematic review and meta-analysis
Source: Public Health Rev. 2026 Jun 25;47:1608819. doi: 10.3389/phrs.2026.1608819 (PMC13345979; doi:10.3389/phrs.2026.1608819)

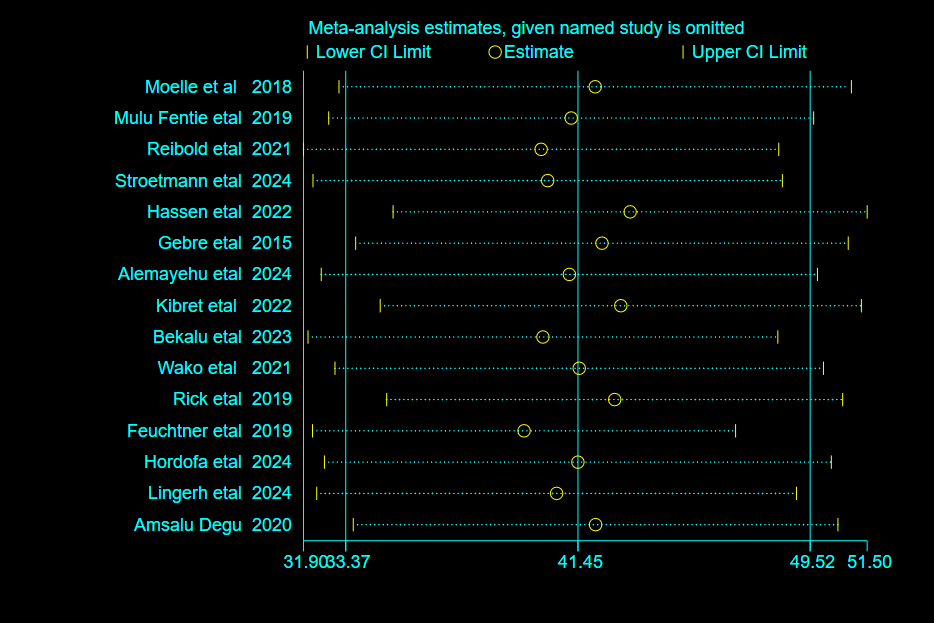

Supplement: Supplementary file 2 [file Image1.TIF]
