# Supplementary material for: Poor adherence to cancer therapy in Ethiopia: systematic review and meta-analysis
Source: Public Health Rev. 2026 Jun 25;47:1608819. doi: 10.3389/phrs.2026.1608819 (PMC13345979; doi:10.3389/phrs.2026.1608819)
Supplement: Supplementary file 3 [file Table2.docx]

**Checklist for analytical observational studies**

Critical Appraisal tools for use in JBI Systematic Reviews

| S.N | **Included studies** | **JBI’s critical appraisal questions** | | | | | | | | Quality  Score | Status |
| --- | --- | --- | --- | --- | --- | --- | --- | --- | --- | --- | --- |
|  |  | Q#1 | Q#2 | Q#3 | Q#4 | Q#5 | Q#6 | Q#7 | Q#8 |  |  |
| 1 | Moelle et al 2018) | N | Y | Y | Y | N | N | Y | Y | 62.5% | √ |
| 2 | Mulu Fentie etal [2019] | Y | Y | Y | Y | N | Y | Y | Y | 87.5% | √ |
| 3 | Reibold etal [2021] | Y | Y | Y | Y | N | N | Y | Y | 75% | √ |
| 4 | Stroetmann etal [2024] | Y | Y | Y | Y | N | N | Y | Y | 75% | √ |
| 5 | Hassen etal[2022] | Y | Y | Y | Y | N | Y | Y | Y | 87.5% | √ |
| 6 | Gebre etal [2015] | Y | Y | Y | Y | N | Y | Y | Y | 87.5% | √ |
| 7 | Alemayehu etal [2024] | Y | Y | Y | Y | N | Y | Y | Y | 87.5% | √ |
| 8 | Kibret etal [2022] | Y | Y | Y | Y | N | Y | Y | Y | 87.5% | √ |
| 9 | Bekalu etal[2023] | Y | Y | Y | Y | N | Y | Y | Y | 87.5% | √ |
| 10 | Wako etal [ 2021] | Y | Y | Y | Y | N | Y | Y | Y | 87.5% | √ |
| 11 | Rick etal [2019] | N | Y | Y | Y | N | N | Y | Y | 62.5% | √ |
| 12 | Fechner etal [2019] | Y | Y | Y | Y | N | N | Y | Y | 75% | √ |
| 13 | Hordofa etal [2024] | Y | Y | Y | Y | N | Y | Y | Y | 87.5% | √ |
| 14 | Lingerh etal [2024] | Y | Y | Y | Y | N | Y | Y | Y | 87.5% | √ |
| 15 | Amsalu Degu[2020] | Y | Y | Y | Y | N | Y | Y | Y | 87.5% | √ |

JBI Critical Appraisal Checklist FORL obsrevational studies

Reviewer_____________________________________Date_______________________________

Author_______________________________________Year_________Record Number_________

| JBI’s critical appraisal questions | Yes | No | Unclear | Not applicable |
| --- | --- | --- | --- | --- |
| 1. Were the criteria for inclusion in the sample clearly defined? | □ | □ | □ | □ |
| 1. Were the study subjects and the setting described in detail? | □ | □ | □ | □ |
| 1. Was the exposure measured in a valid and reliable way? | □ | □ | □ | □ |
| 1. Were objective, standard criteria used for measurement of the condition? | □ | □ | □ | □ |
| 1. Were confounding factors identified? | □ | □ | □ | □ |
| 1. Were strategies to deal with confounding factors stated? | □ | □ | □ | □ |
| 1. Were the outcomes measured in a valid and reliable way? | □ | □ | □ | □ |
| 1. Was appropriate statistical analysis used? | □ | □ | □ | □ |

Overall appraisal: Include=15 Exclude Seek further info=0 □

Comments (Including reason for exclusion)

**Assessment of certainty of the evidence using GRADE**

**Outcome: Poor adherence to cancer therapy in Ethiopia**

| **Domain** | **Assessment** | **NOTES** | **Certainty After Downgrade/Upgrade** |
| --- | --- | --- | --- |
| **Study design** | Low concern | **No-Downgrade** for study design | High |
| **Risk of bias** | Low concern | **No Downgrade.** All the included studies were with low risk of bias | High |
| **Inconsistency** | Very serious (Heterogeneity 98%) | **No Downgrade.** The initial heterogeneity of 98.0% was high, indicating substantial variation in the results across studies. This heterogeneity was reduced, fully explained and resolved through Subgroup and sensitivity analyses. | High |
| **Indirectness** | No serious concerns | **No Downgrade.** Thi study directly address the outcome of interest (adherence to cancer therapy) based on the PICO question of the evidence. | High |
| **Imprecision** | No serious concerns | **No Downgrade.** The study obtained statistically significant (95% CI: 33.37–49.52) suggests that the CI is sufficiently narrow to draw meaningful conclusions about the prevalence of poor adherence. | High |
| **Publication Bias** | No evidence of publication bias; small study effects observed | **No Downgrade.** The analysis indicates "no publication bias and small study effect," which suggests that the published literature is likely to be representative of all studies, and smaller studies do not appear to be unduly influencing the pooled estimate in a biased manner. | High |
| **Overall Certainty of Evidence** | | | High |
